# Commentary: Cortical responses to salient nociceptive and not nociceptive stimuli in vegetative and minimal conscious state

**DOI:** 10.3389/fnhum.2015.00657

**Published:** 2015-12-08

**Authors:** Antonino Naro, Rocco S. Calabrò

**Affiliations:** Behavioral and Robotic Neurorehabilitation Laboratory, IRCCS Centro Neurolesi “Bonino-Pulejo,”Messina, Italy

**Keywords:** pain perception, LEP, DOC, VS, MCS

We read with great interest the work by de Tommaso et al. (2015) assessing cortical responsiveness to nociceptive and multimodal sensory stimuli in patients affected by chronic disorders of consciousness (DOC). In their valuable work, the authors attempted to assign each patient to the most appropriate DOC category by using auditory, visual, somatosensory, and nociceptive laser stimuli. Interestingly, the motor responsiveness to nociceptive stimuli assessed through the Coma Recovery Scale-Revised and the Nociception Coma Scale-Revised correlated with each evoked response, but Laser Evoked Potentials (LEPs). The latter were recognizable in all of the patients, independently from the preservation of other sensory modalities and the motor responsiveness to nociceptive stimuli. Hence, LEP absence should not be considered as a demonstration of the inability to experience pain, since the preservation does not *per se* indicate a conscious pain perception in DOC (de Tommaso et al., 2015). However, it is to note that we should be aware in interpreting authors' findings since these are based on a very limited number of patients (i.e., only 4 MCS and 5 VS). Moreover, since the presence vs. absence of LEPs was based on a subjective visual identification of the event-related potential waveforms, the possibility of misinterpretations (mainly due to very low signal-to-noise ratio) should be taken into account.

Conscious pain perception in DOC patients is a very thorny matter of ethical and clinical debate. Indeed, it is not easy to clinically assess pain perception in the vegetative state (VS) (characterized by non-conscious reflexive behavioral patterns) and minimally conscious state (MCS) patients (characterized by reproducible but fluctuant conscious behavioral patterns), although several clinical scales have been *ad hoc* employed to improve diagnostic accuracy (Chatelle et al., 2012). In addition, functional neuroimaging studies have shown that some DOC individuals can show residual complex brain activations that do not correspond to their behavioral output (e.g., Kassubek et al., 2003; Boly et al., 2008). Thus, a potential pain experience should be considered even in VS individuals, independently from their communication skills.

Although LEPs are commonly used when studying nociceptive pathways, their role concerning pain assessment in DOC individuals could be at first glance limited by the following issues: (i) the basic LEP parameters, including amplitude and latency, cannot give unique information concerning conscious pain perception in DOC patients (de Tommaso et al., 2015); (ii) LEPs mainly constitute a marker of relevant-stimulus dependent arousal (Mouraux and Iannetti, 2009); (iii) LEPs depend on the selective activation of thermo-nociceptive afferents; and (iv) LEP reflect the activity of multiple cortical assemblies within different cortical areas that process either nociceptive or somatosensory inputs (Garcia-Larrea et al., 2003). Nevertheless, the presence or the absence of LEPs could be somehow useful in determining whether or not a patient has the ability to experience pain, although LEPs are not considered a specific marker of pain perception. To this end, some correlations between perceived pain intensity and single-LEP features have been documented (Iannetti et al., 2005; Huang et al., 2013).

In our opinion, further LEP protocols should be fostered in an attempt to more reliably assess pain-perception in DOC patients and reach a more reliable differential diagnosis. In fact, recent studies in healthy individuals have suggested that LEP-related γ-band oscillatory activity (GBO) may have a role in modulating pain intensity within primary somatosensory cortex (Gross et al., 2007; Zhang et al., 2012). It has been recently showed that fronto-parietal GBO and the associated sensory-motor integration processes may reflect the functionality of the fronto-cingulate-parietal network involved in pain perception and pain-gating processes at cortical level, independently from the patient's ability to communicate (Naro et al., 2015a,b).

Unfortunately, GBO analysis presents some limiting factors, including low signal-to-noise ratio, high inter-individual variability, and contamination of ocular movements and/or muscle artifacts. Nonetheless, the evaluation of LEP-related GBO might contribute to uncover residual pain perception even in those totally non-communicative patients, even though it is still debated whether or not GBO can be reliably assessed at single-subject level.

Future studies should be fostered in an attempt to shed some light on the role of combined LEP-GBO analysis in pain perception assessment in DOC patients.

## Conflict of interest statement

The authors declare that the research was conducted in the absence of any commercial or financial relationships that could be construed as a potential conflict of interest.
